# Adolescent digital emotion regulation

**DOI:** 10.1111/jora.13009

**Published:** 2024-08-09

**Authors:** Tom Hollenstein, Katie Faulkner

**Affiliations:** ^1^ Department of Psychology Queen's University Kingston Ontario Canada

**Keywords:** adolescence, digital technology, emotion regulation

## Abstract

The maturation of effective emotion regulation (ER) skills is a core achievement of adolescence and youth are now developing their ER habits and skills in a hybrid reality of digital and non‐digital experiences. We present a new model of adolescent digital emotion regulation as a conceptual framework to help guide burgeoning research in this area. We distinguish two primary processes: the regulation of emotions that have been elicited within digital contexts (i.e., the *regulation of digitally induced emotions*), and how youth regulate their emotions through digital means (i.e., *digitally regulated emotion*). Following the explication of different pathways in the model and consideration of the affordances of digital contexts, we highlight how this framework connects to theory and guides future research.

## INTRODUCTION

The maturation of effective emotion regulation (ER) skills is a core achievement of adolescence (Bradley, 2000; Gross & Thompson, 2007; Lennarz et al., 2019; Silvers, 2022). The combination of cognitive, biological, and social changes endemic to adolescence elicits emotions at greater frequency and intensity than in late childhood (Allen & Sheeber, 2008; Collins & Laursen, 2004; Larson & Ham, 1993; Maciejewski et al., 2015; Rosenblum & Lewis, 2003; Silk et al., 2003) due to at least three co‐occurring phenomena: a rise in novel conditions (e.g., pubertal changes, romantic interests), the intensification of preexisting ones (e.g., social re‐orientation toward peers, academic challenges), and individual differences in how emotions are regulated (e.g., developmental timing and range of regulatory strategies). Moreover, the heterogeneity in how adolescents regulate these emotions (Cracco et al., 2017; Hollenstein & Lougheed, 2013; van Lissa et al., 2019; Zimmermann & Iwanski, 2014, 2018) is related to various aspects of their psychosocial functioning and well‐being (Aldao et al., 2015; Bonanno et al., 2004; De France & Hollenstein, 2017; Lougheed & Hollenstein, 2012). Developmentally, there may not be an increase in the strategies *available* (Zimmermann & Iwanski, 2014, 2018), but there is a growing *capacity* for improved regulation (Fombouchet et al., 2023; Silvers, 2022). Thus, a primary challenge for adolescents is to navigate their newly intensified emotions through developing more effective and situationally contingent regulatory habits (Hollenstein & Lougheed, 2013).

Over more than a decade, however, the circumstances in which adolescents experience their emotions and develop their regulatory skills have expanded beyond face‐to‐face (i.e., “real” world) social contexts. The rise of digital devices and social media has altered the developmental context of all children (Moreno & Uhls, 2019; Odgers & Jensen, 2020; Prinstein et al., 2020), and, because of their greater access and normative social interests, adolescents have been particularly immersed in these platforms (Granic et al., 2020; Orben et al., 2020; Rideout & Robb, 2018; Vogels & Gelles‐Watnick, 2023). The arrival of these new contexts, and the experiences they afford, has challenged the scope and range of existing developmental frameworks (Granic et al., 2020; Navarro & Tudge, 2022). Does the digital landscape introduce entirely novel phenomena to the process of socioemotional development or do these emergent digital contexts provide variation in known processes (i.e., do we need new theories or just apply existing ones)? Consistent with some emerging theoretical models (e.g., co‐construction theory, Ehrenreich et al., 2020; Subrahmanyam et al., 2006), we maintain that the *processes* involving emotional experiences within social contexts, along with the competencies arising from those experiences including ER, remain unchanged. However, the *means* through which these processes are activated now also include digital contexts (Hollenstein & Colasante, 2020).

The purpose of the present paper is to delineate how the normative maturation of adolescent ER can be situated within a digital experience framework. Specifically, we present a model of Adolescent *Digital Emotion Regulation* as a framework for understanding the digital experiences of modern youth for developmentally oriented research. Inspired by the identification of digital ER outlined by human‐computer interaction researchers (Tag et al., 2022; Verma et al., 2023; Wadley et al., 2020), our model differentiates two ways that digital ER may occur. First, we consider the regulation of emotions that have been elicited within digital contexts (i.e., the *regulation of digitally induced emotions*). Second, we consider the affordances of digital contexts within which youth regulate their emotions (i.e., *emotion regulated* via *digital means*). We conclude with how our adolescent digital emotion regulation model may be integrated with two prominent theories of adolescent emotional development, followed by implications and recommendations for future research that will be necessary to understand how, when, and how well adolescents are engaged in digital ER.

Before delineating the model, however, we wish to outline the scope and intent of the present paper. First, the essentials of the model are not exclusive to adolescence—the pathways and processes described could be applicable across the lifespan. Nonetheless, we have chosen the debut of this model to be focused on adolescence because (a) this is currently the age in which digital experiences, particularly social media, rise precipitously (Nesi et al., 2023; Rideout et al., 2022); (b) this is also the age period in which the co‐regulatory effects of a decade of parenting and other influences emerge more clearly as autonomous regulatory habits (Morris et al., 2007, 2017); and (c) adolescence is clearly the age period of greatest concern about how youth are emotionally impacted by digital experiences, yet to date there has been no formal modeling of the regulatory processes involved.

Second, consistent with other models (e.g., Transformational Framework, Nesi et al., 2018b), this is a universal or generic model “agnostic with regard to the benefits or drawbacks” (Nesi et al., 2018b, p. 25). As such, we expect that the underlying emotional and regulatory processes involved are common to all youth. The great advantage of such a framework is that the undeniable and inevitable individual differences in how, when, and for whom emotions are generated and regulated, both digitally and nondigitally, can be compared across ages, social demographics, diverse identities, diagnostic categories, situations, or any other grouping or covariate, as well as in relation to various forms of socioemotional functioning and well‐being. Throughout the paper, and particularly in the concluding sections on integration with theory and recommendations for research, we will highlight some of these possible developmental and between‐group variations.

## A MODEL OF DIGITAL EMOTION REGULATION

**FIGURE 1:**
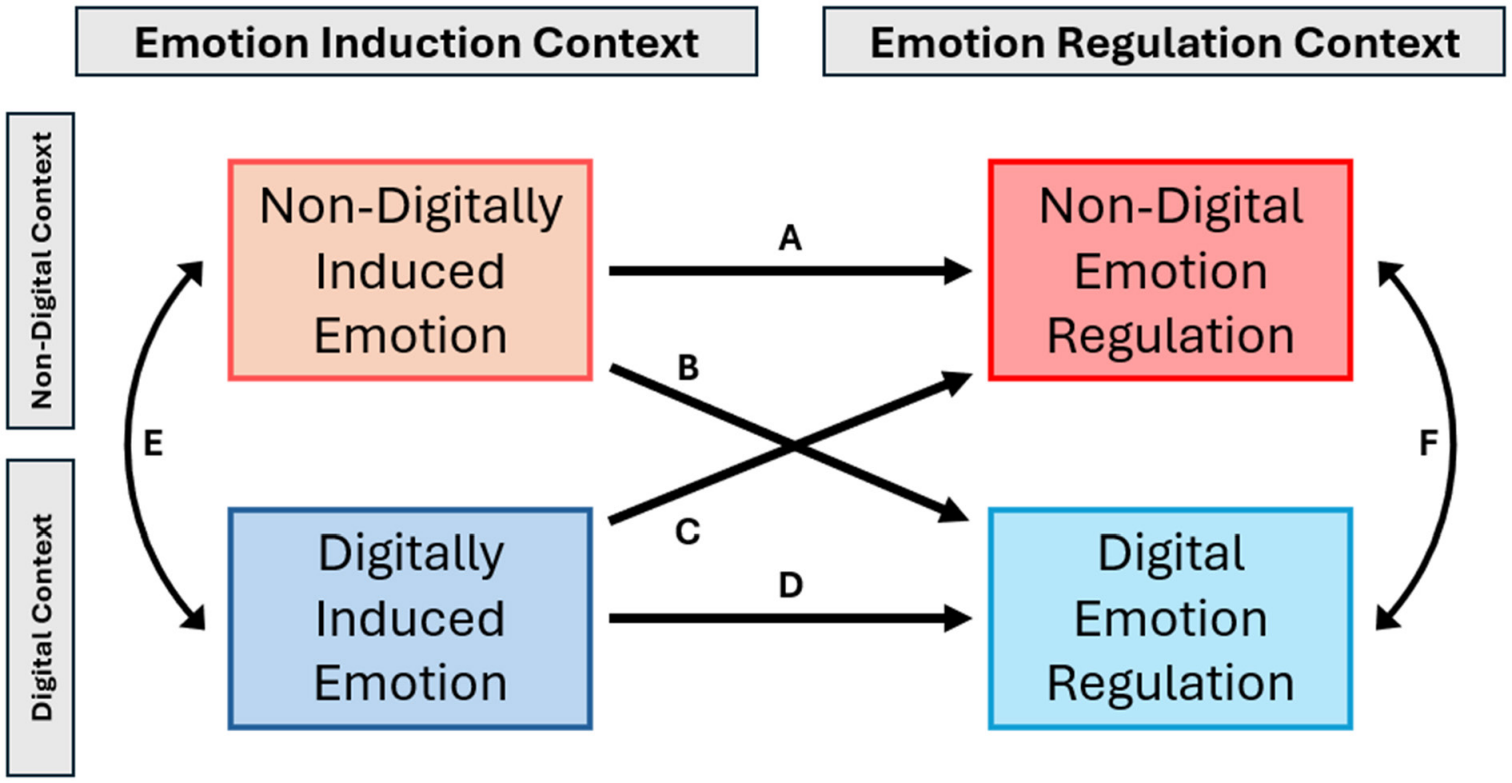
The digital emotion regulation model. Possible connections between emotion generation and its regulation in digital and non‐digital contexts.

Figure 1 depicts our proposed model. The left side of the figure considers whether the emotion in question was induced, elicited, or generated within a nondigital (e.g., face‐to‐face) or digital (e.g., via text message) context. The right side represents the context of regulatory efforts to manage emotions, either nondigital (e.g., self‐generated reappraisal when not engaged with a digital device) or digital (e.g., distracting from an emotion by playing a video game). Pathways A–D represent the possible combinations of digital and non‐digital emotions and their regulation, and pathways E and F represent possible associations between digital and non‐digital emotional experiences and their regulation efforts. We will consider each pathway in turn.

### Old‐school ER: Path A

To date, almost all adolescent, and adult, ER theory and research has focused on Path A. As there are other excellent recent reviews of the adolescent ER literature (e.g., Fombouchet et al., 2023; Silk, 2019; Silvers, 2022), we will not provide a comprehensive overview here. Instead, we highlight certain features relevant to this Path A process that may be pertinent to subsequent considerations of the model.

First, despite recommendations to the contrary (e.g., Cole et al., 2004), most ER research does not account for the emotions that are being regulated. This research usually takes the form of self‐reported frequency of one or two strategies, typically reappraisal and suppression, and finding associations with variables of psychosocial functioning, mental health, or well‐being (Gross, 1998a; Gross & John, 2003; Gullone et al., 2010; Kalokerinos et al., 2015; Richards & Gross, 1999). When emotion types or intensity are considered, such as with experience sampling methods, there is a great deal of variability in when strategies are used and how effective they are (De France & Hollenstein, 2022; English et al., 2017; Haines et al., 2016; Lennarz et al., 2019; McKone et al., 2024; Silk et al., 2003; Tan et al., 2012; Webb et al., 2012; Wylie et al., 2022, 2023). For example, adolescents suppress their emotional expressions more with peers than with parents (Wylie et al., 2023) and are less successful at down‐regulating their negative emotions when they suppress compared to other strategies (De France & Hollenstein, 2022; Lennarz et al., 2019). Moreover, when reported, the multilevel models appropriate for nested experience sampling data typically find that 50–90% of the variance in adolescent emotions and their regulation is at the within‐person level (e.g., Benson et al., 2019; De France & Hollenstein, 2022; McKone et al., 2024). This means that youth are discriminating which ER strategies to implement based on the characteristics of the moment.

The second general point, which is relevant not just to Path A in our model but ER in general, is that the field has suffered from a lack of specificity. Some of this has been due to ambiguity or overinclusiveness about what is being regulated (e.g., feelings, moods, situations), sometimes to the point that any behavior that is initiated in the presence of an emotion is deemed regulatory. Instead, as argued elsewhere (e.g., De France & Hollenstein, 2017), we consider two minimum criteria. First, the regulatory effort or strategy must target an emotion specifically with the intent (not necessarily success) to modulate it. This necessarily excludes mood, which occurs at longer time scales and functions to make specific emotions more or less probable (Lewis, 2000) as well as situations, or problems that persist over time (e.g., a bad relationship). The second criterion is that the regulatory effort has a direct influence over one (or more) of the primary components of an emotion: cognitive appraisal, behavioral expression, and/or psychophysiological arousal. For example, reappraisal targets the cognitive appraisal component, expressive suppression the behavioral expression component (Gross, 1998b, 2015), and direct attempts of relaxation (e.g., deep breathing) targets physiological arousal (De France & Hollenstein, 2017, 2019).

Third, detailed research on the normative, within‐person development of ER across adolescence has been scant. Even more rare has been consideration of how these ER trajectories might mature in different ways across cultures or underrepresented groups (for exceptions see Criss et al., 2021; Teuber et al., 2023). Thus, we have little understanding of the developmental trajectories of long‐standing non‐digital ER (i.e., Path A in the model) in either over‐ or under‐represented populations.

Finally, for a decade or more, adolescent ER‐focused research has not considered digital versus non‐digital contexts. As a result, despite an increase in adolescent ER research in this century, much of what we have come to understand about regulatory habits of youth has either neglected digital experiences or assumed non‐digital contexts. Thus, it is yet unclear as to what may or may not conform to past findings once digital experiences are accounted for. Given that youth are now “constantly online” and all but a few have cell phones or access to social media (Vogels et al., 2022), this gap in our understanding is considerable.

### Digitally regulated emotion: Path B

The second pathway to consider stems from scenarios in which an adolescent has an emotion‐inducing experience in a non‐digital context (e.g., bad grade on a paper, face‐to‐face conflict with a parent, offensive or derogatory comment by a peer) but then attempts to use digital means to regulate it. Examples would include support seeking through texting or social media or distraction through immersing in a video game. These examples raise an important distinction not yet made for our proposed model. While there is necessarily overlap between classic, non‐digital ER and digital ER efforts, there are also unique affordances and constraints for each.

Based on the ecological approach of Gibson (1979), the concept of technological affordances refers to the opportunities that “enable or constrain (but do not determine)” (Manago et al., 2020, p. 154) actions or engagement with a given technology (Hutchby, 2001). When applied more specifically to the recent technological developments that provided the hardware and software for mobile computing and communication opportunities (what we are collectively identifying as “digital” experiences), digital affordances have replicated and/or extended many of the experiences that predate their arrival (Nesi et al., 2018a; Subrahmanyam et al., 2006; Valkenburg, 2022). For example, emotions have been generated and regulated via older interpersonal media, such as letters and telephones, as well as recreational activities, such as playing sports and games. The question then is what is unique to digital ER. Here, we highlight enhancements and limitations of ER in digital contexts based on time and location.

Unlike non‐digital experiences, users can transcend time and space via digital means: expressing emotion or engaging in social support processes can occur asynchronously and/or with someone on the other side of the planet (Colasante et al., 2022; Ellis, 2019; Leick, 2018; Shi et al., 2023; Valkenburg, 2022). Although this extends regulatory opportunities for youth, in some ways it can make digital ER research more complex. Using the social support example, temporal separation of support seeking and support receiving allows for greater clarity of these as separate processes on the one hand. On the other hand, it then becomes difficult to identify when regulation or specific regulation processes were enacted. Thus, the distinctive features of asynchrony and distance in digital ER require careful consideration in research designs, measures, and interpretations, as they present both opportunities and challenges for understanding the regulatory processes involved.

Digital affordances may enhance some specific regulatory efforts more than others. Asynchronous digital activities like texting or scrolling do not require immediate reactions, as when face‐to‐face, but instead enable at least a moment's reflection (Valkenburg, 2022). As such, ER based on cognitive appraisals (e.g., reappraisal and rumination) may be more probable during these activities than in non‐digital contexts. As a second example, video games may be motivated by goals of distraction and avoidance, or they may provide extended opportunities to develop and hone ER skills. Although some games have been developed specifically for enhancing ER, such as MindLight (Schoneveld et al., 2016, 2018, 2019, 2020; Tsui et al., 2021; Wols et al., 2018, 2021), most games often require sustained, focused attention. Hence, redirected focus may prevent attending to or otherwise regulating the precipitating emotion. Alternatively, being able to direct expressions of emotions like anger or activate reconstructed appraisals of the precipitating event may also be functional. There is also the possibility that the degree of cognitive demand in a video game (e.g., puzzle vs. first‐person shooter games) may play a role in distinguishing when or who engages in each of the processes Nonetheless, it is not yet clear how much or in what ways day‐to‐day adolescent digital experiences facilitate particular ER strategies.

There are also limits to some digital ER options, mostly due to affordances of interpersonal synchrony. For example, because expressive suppression tends to occur in the presence of others (e.g., Wylie et al., 2023), digital contexts that lack synchronous observation of one's emotional expressions (e.g., chat that does not include video) are unlikely to be regulated via suppression. According to Social Baseline Theory, ER is less challenging or effortful when in the presence of other people, especially those who feel close (Beckes & Sbarra, 2022; Coan & Sbarra, 2015; Lougheed et al., 2016). Because modes of digital experiences vary in social degree as well as interpersonal synchrony, it is possible that they also differ in the degree of effort necessary to self‐regulate. Social media can convey a strong sense of interconnectedness (Anderson & Jiang, 2018; Orben et al., 2020) as well as emphasize one's separateness from others (Kurten et al., 2023; Livingstone & Helsper, 2007); hence, it is possible that this variation contributes to an adolescent's digital ER effort and success in the moment as well.

In summary, ER can be accomplished through exclusively non‐digital means (e.g., relaxation via deep breathing), exclusively digital means (e.g., asynchronous social support), or, perhaps most often, either digitally or nondigitally (e.g., expressive suppression face‐to‐face or on a video call). The degree to which youth are relying on digital methods for ER is almost completely unknown. However, in our nascent research, we have preliminary evidence that adolescents (aged 13–15) regulated their emotions digitally more than their mothers and more during the first year of the pandemic relative to the year prior (Faulkner et al., 2023). Thus, we have initial confirmation that youth are indeed regulating digitally, but far more work is needed to examine this robustly.

So far, we have considered the regulation of digitally elicited emotions (Path A of Figure 1) and regulation via digital means (Path B of Figure 1). Some of these points may also be salient for the other paths in Figure 1, although there are additional points that will be addressed in the next two sections.

### Regulation of digitally induced emotions: Paths C and D

Digitally induced emotions are not new emotional states, but historically novel circumstances in which these emotional states may be elicited. However, it is possible that previously typical rates and intensities of specific emotions may have changed. As with our consideration of enhancements and limitations of ER strategies above, it may be that digital contexts enhance and limit specific emotions as well. The affordances of social media, for example, have enabled more extensive or more personal experiences of social comparisons (Meier & Johnson, 2022; Vogels et al., 2014) or discrimination (Tynes et al., 2020). Thus, an untested possibility is that occurrences of self‐conscious emotions like shame have increased or that the normative rise of shame proneness in adolescence (Tangney & Dearing, 2002) occurs at earlier ages (Hollenstein & Colasante, 2020). The prevalence of digital experiences that elicit negative emotions (Hamilton et al., 2021), perhaps especially for vulnerable or marginalized youth, means that effective regulation is particularly critical. The degree to which youth choose non‐digital or digital means to achieve that regulation remains unknown, yet we can draw upon recent research about how youth select and deploy various ER strategies.

Strategy choice, flexibility, and situation specificity are growing considerations in recent models of ER (Aldao et al., 2015; Battaglini et al., 2022; Bonanno & Burton, 2013; English & Eldesouky, 2020; Haines et al., 2016; Kalokerinos & Koval, 2022), with some examination in youth samples (De France & Hollenstein, 2022; McKone et al., 2024). These approaches share a central tenet that effective ER is a process of applying specific strategies to fit socioemotional characteristics of the situation (e.g., emotion type, social context, intensity). Because virtually all youth have ready access to digital devices (Rideout et al., 2022; Vogels et al., 2022) and adolescents exercise emotional “niche picking” (i.e., selecting situations and strategies based on their emotional strengths and limitations; Saarni et al., 2008; Morris et al., 2007), we propose that the opportunity to regulate can also include *digital ER niche picking*: the flexible selection of digital and/or non‐digital means for regulation. For example, an adolescent who just received an upsetting text could regulate their distress through soliciting social support from a person in the room with them or through texting a person in another location. Another possible example is using distraction as a prominent strategy with digital devices but using suppression more in non‐digital circumstances. Thus, ER niche picking (Morris et al., 2007) and ER flexibility (Aldao et al., 2015; Battaglini et al., 2022; Bonanno & Burton, 2013; English & Eldesouky, 2020; Haines et al., 2016; Kalokerinos & Koval, 2022) may be extended into digital contexts.

The open question as to what ways ER choice (e.g., Sheppes et al., 2011), flexibility (e.g., Bonanno & Burton, 2013), or situational specificity (e.g., Haines et al., 2016) manifest in or are influenced by digital options may be informed by developmentally informed social media theories. Transactional theories of digital habits emphasize the degree to which users choose specific platforms or experiences based on the needs of the moment (Valkenburg, 2022). Thus, on the one hand, the wider range of regulatory options could enhance flexible adaptation to the specifics of the emotional moment. Youth in the digital age may simply have more, and more precise, options available for regulating their emotions. However, on the other hand, the Transformational Framework (Nesi et al., 2018a) suggests that exercising such ER niche picking options in the digital realm may also be transformative. If youth find digital ER less challenging, they may become less flexible by avoiding more difficult alternate digital or non‐digital options, relying instead on a select few. For example, asynchronous interactions can be less spontaneous (Valkenburg, 2022), allowing for delayed and more controlled expression and regulation. It is possible that this degree of control also limits opportunities for spontaneous in‐person regulation and therefore delays development of critical regulatory skills.

### Within‐adolescent associations between digital and non‐digital: Paths E and F

We have considered how youth might regulate digitally induced emotions and how they might regulate their emotions digitally. Given the possibility of between‐person variation in ER flexibility mentioned above, perhaps a more basic initial question is whether there are any between‐ or within‐person differences across these contexts. One possibility is that ER is more trait like, so regulatory patterns are consistent across both contexts. For example, a youth who relies heavily on distraction to regulate their emotional experiences might not show much difference between digital and non‐digital contexts. ER repertoire research has shown that there is a consistent, albeit small, portion of youth who report reliance on one strategy at the exclusion of all others (De France & Hollenstein, 2017; Lougheed & Hollenstein, 2012; Grommisch et al., 2020; Tan et al., 2022). However, given the evidence from the experience sampling studies that show youth are sensitive to features of the emotional context reviewed earlier (De France & Hollenstein, 2022; Lennarz et al., 2019; Wylie et al., 2023), it is more likely that youth are flexibly adapting their digital ER to the affordances of the moment. To date, however, the relative reliance on digital versus non‐digital ER is unknown. Therefore, trait (i.e., between‐person) versus state (i.e., within‐person) variation should be a central theoretical and empirical focus for digital ER researchers.

Another possibility is that this contrast of digital versus non‐digital ER is only salient to those who grew up without current digital affordances (Livingstone & Byrne, 2018). In their update of Bronfenbrenner's Neo‐ecological Theory (Bronfenbrenner & Morris, 2006), Navarro and Tudge (2022) argue that the developing child exists in multiple microsystems, both virtual and nonvirtual (i.e., “real life”) simultaneously (Navarro & Tudge, 2022). More specifically, for youth these microsystems are indistinguishable as a *hybrid reality* of “an offline world that is woven dynamically and interactively with online contexts in a single holistic ecosystem” (Granic et al., 2020, p. 196). As new technology continues to push developmental contexts further into hybrid realities, this gap between what parents and adults understand from their own childhood experiences and what their children experience may likely continue as well. If so, then this tendency to compare the new with the less new will persist for the older generation (Orben, 2020b), but appear to be a nonsensical distinction for youth. Alternatively, it is possible that youth developing within the past decade retain an appreciation of this hybrid reality when they become parents over the coming decades, rendering the digital versus non‐digital distinction less important. Thus, it remains to be seen whether it may become necessary to update and further contextualize the pathways of our model.

## INTEGRATION WITH DEVELOPMENTAL THEORY

As described earlier, our digital ER model, on its own, is not specifically an adolescent model and pathways likely vary by age and developmental stage. Now that the model has been explicated, we wish to explore how it can be situated in the maturational flow from childhood to adulthood. To do so, we discuss two prominent and emotionally relevant developmental theories: developmental neuroscience models and the tripartite model.

### Neural systems and digital ER

Neural models of adolescent development are based in normative maturational patterns of brain and concomitant behavior (e.g., Casey, 2015; Dahl, 2004; Somerville, 2013; Steinberg, 2008). Though more elaborate than oversimplified “dual systems” conceptualizations (Casey et al., 2016; Pfeifer & Allen, 2012), the basic observations across models are the same: early adolescence (roughly 10–15 years old) is characterized by a biologically mediated surge in emotionality and amplified orientation toward peers (Nelson et al., 2016), which is eventually followed by greater capacities of self‐regulation in later adolescence. As others have noted (Hamilton et al., 2022; Nesi et al., 2018a, 2018b), this developmental timing makes younger adolescents particularly more interested in digital experiences, particularly social media, though perhaps less capable in regulating both their digital habits and the emotions that arise in digital contexts. In terms of our model, this would suggest that the methods of digital ER as well as the efficacy of those efforts might differ between early and late adolescence. One possible result could be that younger, compared to older, adolescents have a more limited repertoire of both digital and non‐digital ER strategies to draw upon, creating different patterns across digital ER pathways. This could limit their situation‐specific flexibility, rendering key differences in pathways C and D, for example. A second hypothesis based on these neural systems accounts might be to expect greater differences between social and non‐social contexts for the younger versus older adolescents. Given sex differences in the timing of pubertal and neural transitions underlying social reorientation and emotionality (Blakemore, 2008; Mendle et al., 2010), we might also predict that the age differences described above might occur earlier for girls relative to boys.

### The tripartite model of emotion socialization and digital ER

The tripartite model of emotion socialization (Morris et al., 2007, 2017) delineates the means through which emotion‐related experiences within the family context (e.g., emotion modeling and coaching) may explain individual differences in children's long‐term developmental outcomes of adjustment, socioemotional functioning, and mental health. The mediating mechanism through which children internalize their socialization experiences and subsequently navigate their own emotional worlds is emotion regulation. Incorporating our adolescent digital ER model into this framework would allow deeper consideration of the mechanisms underlying the development of digital ER in at least two ways.

First, the primary parenting processes of direct socialization of emotion (i.e., coaching, scaffolding, support) now include children's emotional experiences in the digital realm. By providing instruction on how to navigate myriad inevitable emotional experiences, caregivers can foster more adept ER as their children grow through childhood and adolescence. However, caregivers must now extend their empathic listening and validation skills to activities and interpersonal experiences that are at least somewhat unfamiliar to them (Modecki et al., 2024). Thus, direct emotion socialization now includes *digital parenting*.

Digital Parenting refers to parenting processes that occur *within* digital contexts (e.g., texting; Ehrenreich et al., 2021) as well as *about* digital habits (e.g., limit setting; Modecki et al., 2024; Odgers, 2019). Within digital contexts, parents can more easily support and monitor their child from afar and share experiences such as games. Thus, parenting processes extend into relatively novel contexts, and due to technological affordances, are not bound by geographic or time constraints. Parents and youth also experience conflicts about digital habits. For example, in our recent sample of 11–12 year‐olds and their mothers, prior to the pandemic, we observed that 97% of mothers and 78% of youth identified digital issues (e.g., phone use, video games) as sources of conflict, with the majority identifying these as their most intense conflicts. Both within and outside of digital contexts, parents continue to guide their adolescent's emotions and behaviors through well‐known practices such as monitoring, rule setting, discipline, and instruction (Pettit et al., 2001) as well as newer practices such as digital co‐use or the application of content filters (Modecki et al., 2024). Furthermore, as with predigital era technology (e.g., TV), caregivers may use digital devices and experiences specifically to regulate their child's emotions from infancy onward (Hassinger‐Das et al., 2020; Radesky, Peacock‐Chambers, et al., 2016). Thus, the current generation of adolescents has had a great deal of instruction in regulating their emotions digitally from a young age.

A second way that the tripartite model may inform the development of digital ER is through indirect emotion socialization processes (i.e., children's observations of family emotions). This would include all of the digital ER processes outlined above in our description of the model. In particular, parents are now modeling digital habits and behaviors for their children and parental digital habits predict subsequent child habits (Lauricella et al., 2015). Digital devices have also introduced a new method for offspring to observe digital habits, such as “technoference,” or the neglect of a child in lieu of a digital device (McDaniel et al., 2018; McDaniel & Radesky, 2018). Frequent technoference may socialize emotions by limiting face‐to‐face, dynamic interactions, providing important signals to children about parental availability and support as well as ways of regulating through digital means.

These are just two examples of how developmental theory, in combination with our digital ER model, can provide a framework for inquiry. The limited but potent observations of the digital experiences of youth and their families over the past decade or so further inform and inspire a litany of research questions that need answering. Does reliance on media ER at early ages have an impact on adolescent digital ER? Given the heterogeneity in parents' and teachers' technology use and attitudes (George & Odgers, 2015; Radesky, Eisenberg, et al., 2016), how does the modeling and coaching of digital emotion regulation by adult socializers intersect with adolescent engagement in digital ER? We suggest an approach to illuminating the emergence and impact of youth digital ER process next.

### Recommendations for future research

The proposed framework is intended to help guide much needed research. There have been excellent papers on how best to conduct research in the digital domain (e.g., Griffioen et al., 2020; Hamilton et al., 2022; Modecki et al., 2024; Odgers & Jensen, 2020; Orben, 2020a, 2020b), so we will not repeat all their recommendations here. Instead, we make a select set of recommendations that are more specifically pertinent for adolescent digital ER.

First, because this is a very new domain of inquiry, the initial scientific steps are necessarily descriptive. In what ways and how often are youth regulating digitally induced emotion and using digital means to regulate? Rather than rush to prediction, prevention, and intervention, the foundation of normative habits and methods must be established. To that end, the development of robust measures is essential. The current quality of the measures used in digital research is generally below well‐accepted standards (Davidson et al., 2022; Griffioen et al., 2021; Kaye et al., 2020; Orben & Przybylski, 2019;). Virtually every current recommendation is to throw out flawed measures such as “screen time” in lieu of more precise measures that capture the functions and platform specific behaviors (Barr et al., 2020; Davidson et al., 2022; Ellis, 2019; Ellis et al., 2019; Granic et al., 2020; Kaye et al., 2020; McFarland et al., 2023; Odgers & Jensen, 2020; Orben, 2020a). Digital ER is just such a function, but valid and reliable self‐report, lab task, and observational measures have yet to emerge. It is only from a robust body of descriptive work on digital ER that it will be possible to understand which individual differences are functional and which may be problematic.

Second, in line with this descriptive approach, the appreciation of emotions, particularly those labeled as negative, as necessary and functional should guide this, and arguably all, ER research. For example, experiencing the emotion of anxiety is normative (Kurth, 2016; Parrott, 2002; Perkins & Corr, 2014) and what distinguishes problematic from non‐problematic anxiety is how it is regulated (Dennis‐Tiwary, 2022). Similarly, negative emotions such as anger and anxiety arising from witnessing acts of social injustice online is normative and may motivate prosocial behavior (Koenig & McLaughlin, 2018). Thus, although the motivation for digital research may be to ascertain psychosocial functioning, mental health, or wellbeing, the interpretations of the mere occurrence of a given emotion requires context and, ideally, process.

Third, to get from description to process, it will be necessary to conduct real‐time and longitudinal research. Ultimately, the inferences we wish to make about ER in general, and by extension digital ER, is how emotions are regulated moment‐by‐moment (Cole & Hollenstein, 2018). Each emotional experience builds upon the next across development, so the real‐time rise and fall of emotions is the bedrock of models at other time scales (Cole et al., 2004). This is why research designs and analytical models must focus on within‐person processes because cross‐sectional, between‐subject correlations obscure the insights and inferences we seek (Hamaker, 2022). In lab observations and experimental manipulations may reveal some of these processes. For example, Griffioen et al. (2021) implemented a clever design of observing participants smart phone use when left alone in the lab following a stress task. Through coding of a video obtained by an overhead camera as well as a stimulated recall interview, they ascertained the apps, engagement (active or passive), interactants, and the feelings of the participants during their 10‐min “waiting” period. Variations of such a design, including other measures of emotional expression and physiological arousal, could be used to elucidate real‐time digital ER processes.

At slightly longer time scales, experience sampling methods can be used to get more detail about youth digital ER in day‐to‐day life. To date, studies that have not differentiated digital from non‐digital contexts have shown that adolescents do select strategies and have varying levels of success across features of their emotional contexts such as controllability and who they were with (De France & Hollenstein, 2022; English et al., 2017; Haines et al., 2016; Lennarz et al., 2019; McKone et al., 2024; Silk et al., 2003; Tan et al., 2012; Webb et al., 2012; Wylie et al., 2022, 2023). It will only take a small addition to these study designs to test all the pathways in our model simultaneously (see Figure 1).

Finally, it will be essential to go beyond basic testing of each of the pathways in the model (Figure 1), preferably all at once, to make explicit comparisons across age, gender, SES, and various identity and diagnostic groups. Do those who rely more heavily on digital experiences or those who are at greater risk for deleterious emotional outcomes exhibit different patterns or strength of pathways in the model? Are there important cultural differences? As with much of non‐digital ER research, more inclusive and non‐W.E.I.R.D. samples are necessary for a complete understanding of these modern phenomena.

## CONCLUSION

Because youth develop with the same needs (e.g., for agency and communion; Granic et al., 2020) as previous generations, their social and emotional development relies on their maturation of intra‐ and inter‐personal emotion regulation across myriad contexts. These contexts now include digital ones (Hollenstein & Colasante, 2020). Here, we have provided a framework for how to consider and integrate both digital and non‐digital experiences into adolescent ER research.

Further, we hope that our approach to providing a framework predicated firmly on developmental science becomes more common in other domains relevant for the study of digital experiences in youth. We have tried to highlight those researchers who are making great strides in this direction (e.g., Ehrenreich et al., 2020; George & Odgers, 2015; Granic et al., 2020; Modecki et al., 2024; Odgers & Jensen, 2020). However, the lion's share of research in this domain comes out of the disciplines of media studies, communication, medicine, and human‐computer interaction. It is time for those who do basic research on developmental processes to integrate the now ubiquitous digital landscape within which youth develop with the vast empirical and theoretical knowledge gained over the past century.

## CONFLICT OF INTEREST STATEMENT

No conflicts of interest to declare.

## Data Availability

There is no data for this paper, therefore no data can be made available.
